# Vitamin D and cinacalcet administration pre-transplantation predict hypercalcaemic hyperparathyroidism post-transplantation: a case-control study of 355 deceased-donor renal transplant recipients over 3 years

**DOI:** 10.1186/s13737-014-0021-5

**Published:** 2014-12-31

**Authors:** Frank-Peter Tillmann, Carolin Wächtler, Anita Hansen, Lars Christian Rump, Ivo Quack

**Affiliations:** Klinik für Nephrologie, Heinrich Heine Universität Düsseldorf, Moorenstr. 5, D-40225, Düsseldorf, Germany

**Keywords:** Cinacalcet, Kidney transplantation, Waiting list, Parathyroid hormone, Hypercalcaemia

## Abstract

**Background:**

The effects of pre-transplantation medication for secondary hyperparathyroidism on post-transplantation parathyroid hormone (PTH) and calcium levels have not yet been conclusively determined. Therefore, this study sought to determine the level of off-label use of cinacalcet and to determine predictors of its administration during the long-term follow-up of a cohort of individuals who received deceased-donor renal transplants. Furthermore, safety considerations concerning the off-label use of cinacalcet are addressed.

**Methods:**

This was a case-control study of 355 stable renal transplant recipients. The patient cohort was divided into two groups. Transplant group A comprised patients who did not receive cinacalcet treatment, and transplant group B comprised patients who received cinacalcet treatment during follow-up after renal transplantation. The characteristics of the patients were evaluated to determine predictors of cinacalcet use after successful renal transplantation.

**Results:**

Compared with the control individuals (*n* = 300), the cinacalcet-treated individuals (*n* = 55) had significantly higher PTH levels at 4 weeks post-transplantation (20.3 ± 1.6 versus 40.7 ± 4.0 pmol/L, *p* = 0.0000) when they were drug naive. At 3.2 years post-transplantation, cinacalcet-treated patients showed higher PTH (26.2 ± 2.3 versus 18.4 ± 2.3 pmol/L, *p* = 0.0000), higher calcium (2.42 ± 0.03 versus 2.33 ± 0.01 mmol/L, *p* = 0.0045) and lower phosphate (0.95 ± 0.04 versus 1.06 ± 0.17 mmol/L, *p* = 0.0021) levels. Individuals in the verum group were more likely to receive cinacalcet therapy (45.5% versus 14.3%, *p* = 0.0000), and they had higher pill burdens for the treatment of hyperparathyroidism (1.40 ± 0.08 versus 0.72 ± 0.03 pills per patient, *p* = 0.0000) whilst they were on the waiting list for transplantation. Regression analysis confirmed the associations between hypercalcaemic hyperparathyroidism and PTH levels at 4 weeks post-transplantation (*p* = 0.0001), cinacalcet use (p = 0.0000) and the preoperative total pill burden (*p* = 0.0000). Renal function was the same in both groups.

**Conclusions:**

Parathyroid gland dysfunction pre-transplantation translates into clinically relevant hyperparathyroidism post-transplantation, despite patients being administered more intensive treatment whilst on dialysis. PTH levels at 4 weeks post-transplantation might serve as a marker for the occurrence of hypercalcaemic hyperparathyroidism during follow-up.

## Introduction

Although the spontaneous resolution of renal hyperparathyroidism after kidney transplantation has been described, persistent hyperparathyroidism with hypercalcaemia is frequently encountered during follow-up in renal transplant patients [1,2]. Hypercalcaemia has been associated with vascular calcifications, nephrolithiasis, tubular microcalcifications and the deterioration of renal transplant function [3]. Parathyroidectomy is an invasive therapy with inherent potential complications, including acute and chronic hypocalcaemia, persistent hypoparathyroidism and subsequent adynamic bone disease. Furthermore, the negative impact of parathyroidectomy on kidney function in transplant recipients has been demonstrated [4]. Accordingly, an effective and safe therapeutic alternative is warranted for renal transplant patients who have persistent hypercalcaemic hyperparathyroidism. Cinacalcet was first approved for use in end-stage renal disease (ESRD) patients on chronic renal replacement therapy in March 2004 in USA and in October 2004 in Germany. It is the only available allosteric activator of the calcium-sensing receptors in the human parathyroid gland [5]. Several studies have reported on its beneficial use in patients suffering from primary hyperparathyroidism [6]. The effects of cinacalcet administered after kidney transplantation have been demonstrated in small retrospective [7-9] and prospective [10-12] clinical studies. However, a major concern is the potential for marked hypercalciuria with nephrocalcinosis following cinacalcet administration once kidney function is restored [13,14]. The use of cinacalcet in renal transplant patients remains off-label because of safety concerns with regard to kidney function.

The aim of this study was to evaluate the level of the off-label use of cinacalcet and to determine predictors of its administration during the long-term follow-up of a cohort of individuals who received deceased-donor renal transplants. Furthermore, safety considerations concerning the off-label use of cinacalcet are addressed.

## Materials and methods

### Study approval

The study was approved by the local ethics committee. Data were coded in a manner that ensured subjects could not be identified either directly or through linked identifiers. The clinical and research activities reported are consistent with the “Principles of the Declaration of Helsinki” [15].

### Study population

We analysed 355 patients who received deceased-donor renal transplants between January 2004 and December 2010. The patients comprised 209 (58.9%) men and 146 (41.1%) women. The average age of the patients at the time of renal transplantation was 53.9 years, and they had been on chronic renal replacement therapy for approximately 6.4 years. The mean follow-up period for the cohort was 3.2 years. The causes of renal failure included chronic glomerulonephritis (34.1%), autosomal-dominant polycystic kidney disease (14.6%), interstitial nephritis and chronic reflux (8.2%), systemic lupus erythematosus (1.1%), diabetes (3.9%), other causes (19.2%) and unknown causes (18.9%). Of the 355 transplant recipients, 298 (83.9%) patients had received their first, 44 (12.4%) patients had received their second, 12 (3.4%) patients had received their third and one (0.3%) patient had received her fourth renal allograft. At the time of their last visits to our out-patient clinic, 254 (71.5%) patients were on tacrolimus-, 86 (42.2%) patients were on cyclosporine-, eight (2.3%) patients were on everolimus- and seven (2.0%) patients were on sirolimus-based immunosuppressive regimens. The patient cohort was divided into two groups. Transplant group A comprised patients who did not receive cinacalcet treatment, and transplant group B comprised patients who received cinacalcet treatment during follow-up after renal transplantation.

### Standard values for routine blood chemistry tests

The standard values used for routine blood chemistry tests were as follows: creatinine < 0.9 mg/dL; calcium 2.10–2.42 mmol/L, phosphate (0.89–1.45 mmol/L), alkaline phosphatase (35–104 U/L) and parathyroid hormone (PTH) (1.6–6.9 pmol/L).

### Data acquisition

All data were extracted from clinical charts or electronic databases, including the hospital's laboratory database and Eurotransplant's electronic resource.

### Statistical analysis

The main “outcome variable” was a “cinacalcet prescription” at the time of the patient's final visit to our out-patient clinic, and we analysed its relationship with several covariates. The continuous variables included: the dialysis vintage (years), age at the time of renal transplantation (years), creatinine concentration (mg/dL), calcium concentration (mmol/L), phosphate concentration (mmol/L), alkaline phosphatase concentration (U/L), parathyroid hormone (PTH) level 4 weeks post-transplantation (pmol/L), PTH level at the end of the follow-up period (pmol/L), estimated glomerular filtration rate (eGFR), determined using the Chronic Kidney Disease Epidemiology Collaboration (CKD-EPI) formula, and the follow-up time (years). The categorical variables included sex, parathyroidectomy, cinacalcet treatment, vitamin D treatment, the total pill burden for the treatment of renal hyperparathyroidism whilst on the waiting list and the immunosuppressive regimen administered.

The variables “parathyroidectomy” and “vitamin D treatment whilst on the waiting list” were defined as total and subtotal operative procedures and medication with plain or active vitamin D derivatives immediately before transplantation, respectively. Descriptive statistics including the means, standard errors and proportions are presented for each variable of interest. The Kolmogorov-Smirnov test was used to determine whether the data were normally distributed. The Mann-Whitney U and the chi-square tests were used to analyse inter-group differences. The Bonferroni method was applied to adjust for the effects of multiple testing, and a *p* value of < 0.0071 was considered statistically significant.

Univariate logistic regression analysis was performed for the continuous and categorical variables of interest to evaluate the predictors of and to determine the risk ratios (RRs) for the outcome parameter, that is, cinacalcet use post-transplantation. For each significant variable, the RR, 95% confidence intervals and *p* values were reported. The statistical analyses were performed using the Statistical Package for the Social Sciences 14.0 for Windows (SPSS Inc., Chicago, IL, USA).

## Results

The characteristics of the patients within the two study groups are presented in Table 1. Approximately 15.5% of our patient cohort was prescribed cinacalcet as an off-label medication during the follow-up period after successful renal transplantation. At 3.2 years post-transplantation, patients who were treated with cinacalcet had higher mean calcium levels (2.42 ± 0.03 versus 2.33 ± 0.01 mmol/L, *p* = 0.0045), lower phosphate levels (0.95 ± 0.04 versus 1.06 ± 0.17 mmol/L, *p* = 0.0021), higher PTH levels 4 weeks after transplantation (40.7 ± 4.0 versus 20.3 ± 1.6 pmol/L, *p* = 0.0000) when they were drug naive, higher PTH levels at the end of the follow-up period (26.2 ± 2.3 versus 18.4 ± 2.3 pmol/L, *p* = 0.0000) and higher pill burdens for the treatment of hyperparathyroidism whilst they were on the waiting list (1.40 ± 0.08 versus 0.72 ± 0.03 pills, *p* = 0.0000), and they were more likely to have been prescribed cinacalcet whilst they were wait listed (45.5% versus 14.3%, p = 0.0000), compared with the patients who had not received cinacalcet. Renal transplant function, which was determined using the CKD-EPI formula, was similar for the two patient groups (44.4 ± 1.2 versus 47.4 ± 2.6 mL/min/1.73 m^2^, *p* = 0.2435).

**Table 1:** **Overview statistical results of continuous and categorical variables of transplant group A (control) and transplant group B (cinacalcet) on follow-up**

	**Transplant group A control (** ***n*** **= 300, 84.5%)**			**Transplant group B cinacalcet (** ***n*** **= 55, 15.5%)**					
**Variable**	**Mean + SE**	**95% CI**	**Range**	**Mean + SE**	**95% CI**	**Range**	***p*** ** value**	**RR**	**95% CI**
Dialysis vintage (years)	6.33 ± 0.19	5.95–6.70	23.62	6.88 ± 0.32	6.24–7.53	11.06	0.0417	-	-
Age at time of RTx (years)	53.67 ± 0.73	52.2–55.1	54.9	55.1 ± 1.3	52.6–57.6	33.7	0.5915	-	-
Creatinine (mg/dL)	1.81 ± 0.04	1.73–1.90	4.80	1.64 ± 0.09	1.45–1.83	3.40	0.0589	-	-
Calcium (mmol/L)	2.33 ± 0.01	2.30–2.35	1.42	2.42 ± 0.03	2.37–2.48	0.97	0.0045	1.2	1.1–1.4
Phosphate (mmol/L)	1.06 ± 0.17	1.02–1.09	1.83	0.95 ± 0.04	0.87–1.03	2.02	0.0021	0.2	0.1–0.7
AP (U/L)	83 ± 3	78–89	545	100 ± 6	78–101	262	0.0608	-	-
PTH 4 weeks post-transplant (pmol/L)	20.3 ± 1.6	17.2–23.5	332	40.7 ± 4.0	32.8–48.7	135	0.0000	1.5	1.2–1.8
PTH on follow-up (pmol/L)	18.4 ± 2.3	13.8–23.0	474.9	26.2 ± 2.3	21.6–30.8	72.4	0.0000^a^	-	-
eGFR (CKD-EPI formula)	44.4 ± 1.2	42.0–46.7	121.6	47.4 ± 2.6	42.1–52.6	86.3	0.2435	-	-
Follow-up after RTx (years)	3.26 ± 0.12	3.03–3.48	8.08	3.50 ± 0.26	2.98–4.03	7.57	0.3641	-	-
Gender (%)	♂ 60.3	♀ 39.7	-	♂ 50.9	♀ 49.1	-	0.2330	-	-
Parathyroidectomy^b^ (%)	Yes 27.3	No 72.7	-	Yes 16.4	No 83.6	-	0.0948	-	-
Cinacalcet use on waiting list (%)	Yes 14.3	No 85.7	-	Yes 45.5	No 54.5	-	0.0000	5.0	2.7–9.3
Vitamin D use on waiting list^b^ (%)	Yes 60.0	No 40.0	-	Yes 65.5	No 35.5	-	0.5481	-	-
HPT pill burden on waiting list^b^	0.72 ± 0.03	0.65–0.79	2	1.40 ± 0.08	1.24–1.56	2	0.0000	5.2	3.1–8.7

The Mann-Whitney U test and Fisher's exact test were used to compare for inter-group differences of continuous and categorical variables (a *p* value < 0.0071 was considered significant). To test for predictors of cinacalcet use after renal transplantation, univariate logistic regression was applied and risk ratios (RRs) of significant variables were calculated. Risk ratios and their respective 95% CI were calculated for increments as follows: calcium for an increase of 0.1 mmol/L, phosphate for an increase of 1.0 mmol/L and PTH 4 weeks post-transplant for an increase of 20 pmol/L. On univariate logistic regression, *p* values of significant variables (not shown in the table) were: 0.0039 for calcium, 0.0124 for phosphate, 0.0001 for PTH at time of RTx, 0.0000 for pill burden and 0.0000 for cinacalcet use on waiting list, respectively.

*SE* standard error, *RTx* renal transplantation, *HPT* hyperparathyroidism.

^a^The variable “PTH on follow-up” was not significant on logistic regression (*p* value 0.2003).

^b^The variables “parathyroidectomy”, “vitamin D use on waiting list” and HPT “pill burden on waiting list” encompass total and subtotal operative procedures, plain and active vitamin D derivatives and pill counts of cinacalcet and vitamin D derivatives, respectively.

Univariate regression analysis to determine the predictors of cinacalcet use post-transplantation showed significant associations with calcium levels (RR = 1.2 for an increase of 0.1 mmol/L, *p* = 0.0039), phosphate levels (RR = 0.2 for an increase of 1.0 mmol/L, *p* = 0.0124), the PTH level at the time of renal transplantation (RR = 1.5 per increase of 20 pmol/L, *p* = 0.0000), an increased pill burden for the treatment of hyperparathyroidism whilst on the waiting list (RR = 5.2 per pill, *p* = 0.0000) and “cinacalcet use whilst on the waiting list” (RR = 5.0, *p* = 0.0000). The immunosuppressive therapy regimens did not differ significantly between the study groups (*p* = 0.7863) (Table 2).

**Table 2:** **Overview of immunosuppressive regimens in absolute numbers and percentages; mTORs = sirolimus and everolimus, chi-square** ***p*** **= 0.7863**

	**Cyclosporin**	**Tacrolimus**	**mTORs**	**Numbers**
Transplant group A (control)	72 (24.0)	216 (72.0)	12 (4.0)	*n* = 300
Transplant group B (cinacalcet)	14 (25.5)	38 (69.0)	3 (5.5)	*n* = 55

## Discussion

Approximately 15.5% of our stable renal transplant recipients had been prescribed cinacalcet during the follow-up period after successful renal transplantations. Cinacalcet-treated patients were administered more rigorous medical regimens for the treatment of renal hyperparathyroidism pre-transplantation, which could be considered a marker for the acquisition of more severe forms of renal hyperparathyroidism whilst patients are on chronic renal replacement therapy. Furthermore, an association between cinacalcet use after transplantation and a reduction in transplant function could not be determined. Hypercalcaemia as a consequence of persistent hyperparathyroidism is frequently encountered in renal transplant patients during their routine follow-up visits, and it can lead to increased risks of osteoporosis, fractures, nephrocalcinosis, pancreatitis, vascular calcifications, hypophosphataemia and a reduction in graft function. The limited utility of vitamin D sterols in the treatment of post-transplantation hypercalcaemic hyperparathyroidism means that cinacalcet represents the only pharmacological approach to the treatment of these patients. In small, non-randomised clinical studies, cinacalcet effectively reduced calcium and PTH levels and it helped to normalise phosphate levels [16]. Cinacalcet allosterically modifies the calcium-sensing receptors on the thick ascending limb of Henle, thereby increasing renal calcium excretion [17]. Several reports have documented hypercalciuria after the introduction of cinacalcet treatment to renal transplant patients [10,18], and some reports have linked hypercalciuria to nephrocalcinosis and a decline in graft function [13,19]. However, other studies have determined that cinacalcet has no effect on renal calcium excretion [11,12]. These disparate findings might be explained by the time-dependent effect of cinacalcet in relation to renal calcium handling, because it increases calcium excretion within the first 8 h of its administration and has no further effects thereafter [20]. Other investigations have also reported on safety issues with respect to the potentially negative impact of cinacalcet therapy on renal graft function [14,21,22], but the decline in graft function is relatively small or even reversible after the cessation of cinacalcet medication, which indicates a hemodynamic effect associated with the drug [23]. In contrast to these findings, other studies could not detect a negative effect of cinacalcet on graft function [24-26]. With respect to graft function, our data are more consistent with the latter reports. After an average follow-up duration of 3.2 years post-transplantation, patients receiving cinacalcet therapy showed an eGFR that was 3.0 mL/min/1.73 m^2^ greater than their untreated counterparts. Nevertheless, despite cinacalcet treatment, PTH levels remained significantly higher than those in the control group, which partially explains the differences in the calcium and phosphate levels between the study groups.

At our institution, medication for hyperparathyroidism is usually paused on the day of transplantation and PTH levels are regularly measured to observe their natural courses. Jadoul and co-workers found that stopping cinacalcet treatment on the day of transplantation had no effect on the courses of the PTH levels during 12-month follow-up periods in 28 patients [27]. In contrast, it has been reported that PTH levels are prone to rebounds short after the cessation of medication in patients who received cinacalcet after transplantation [28,29]. In our study, PTH levels differed significantly between the groups as early as 4 weeks after transplantation, which concurs with the latter investigations. Furthermore, we were able to demonstrate a tendency towards reduced parathyroidectomy rates in our study population, as demonstrated in patients with ESRD on dialysis [30]. The dialysis vintage was similar in the two study groups, with a tendency towards longer ESRD treatment periods in the cinacalcet group. Recently, the investigators associated with the Evaluation of Cinacalcet Hydrochloride (HCl) Therapy to Lower Cardiovascular Events study, which was a placebo-controlled prospective study of over 3,800 ESRD patients on haemodialysis in an unadjusted intention-to-treat analysis, reported that cinacalcet had no effect on cardiovascular endpoints [31].

In contrast to these data, a report from a large cohort study of over 100,000 ESRD patients on haemodialysis suggests that improvements in the control of phosphate and PTH levels are associated with improved patient survival [32]. However, these results might not be transferrable to a transplant setting given the differences in the characteristics between ESRD patients on haemodialysis and transplant recipients during follow-up. As a consequence of the retrospective design of our study, we were unable to address the safety issues that are related to potential drug interactions between cinacalcet and standard immunosuppressive therapeutics, including calcineurin and mammalian target of rapamycin inhibitors. Since we could not determine any differences between the two study groups with respect to the standard immunosuppressive drug regimens, we can infer that cinacalcet does not have negative impacts on the standard immunosuppressive drug regimens. Furthermore, Evenepoel and co-workers did not report any negative drug-to-drug interactions in a prospective multi-centre study [33]. Analysis of the data from the pre-transplantation period and the follow-up data, shows that poorly controlled hyperparathyroidism as a consequence of gland hyperplasia in haemodialysis patients before transplantation, is not necessarily resolved by renal transplantation, resulting in poorly controlled hyperparathyroidism for several years after transplantation; hence, our data indicate that parathyroid gland hyperplasia with concomitant hyperparathyroidism is transferred and that it lingers into the post-transplantation era in kidney graft recipients.

Furthermore, hypercalcaemic hyperparathyroidism occurs in a key subset of long-term kidney transplant recipients. More research into optimal PTH ranges in transplant candidates who are on waiting lists is necessary to enable the transplant community to achieve better control of PTH levels post-transplantation. Although prescribing cinacalcet to transplant patients seems to be safe with regard to transplant function, studies that investigate its use with robust endpoints are clearly warranted to shed more light on the long-term usefulness of cinacalcet after renal transplantation.

### Limitations and strengths

There are several limitations to this study. Our study results clearly require corroboration from investigations with larger numbers of patients. Given the retrospective nature of our analysis, we could not account for changes in medications between the two time points. Finally, our data were derived from a single centre and from transplant recipients whose genetic backgrounds were from Caucasian ancestries. Accordingly, these results might not be applicable to other transplant centres or to patients with different genetic backgrounds. The key strengths of our study included the long follow-up period after renal transplantation, the presence of a control group and the availability of a combination of pre- and post-transplantation data.

## Conclusion

PTH levels at as early as 4 weeks post-transplantation are associated with the evolution of hypercalcaemic hyperparathyroidism during long-term follow-up assessments after kidney transplantation. Despite more intensive treatment of renal hyperparathyroidism with cinacalcet and vitamin D derivatives whilst patients are on dialysis, the development of more severe forms of post-transplantation hyperparathyroidism that subsequently affect calcium and phosphate levels continues to occur in a substantial number of patients. The off-label use of cinacalcet is not associated with a decline in transplant function. Further research into the impacts of treatment algorithms for renal hyperparathyroidism associated with wait-listed ESRD programmes on outcome parameters post-transplantation is warranted.
